# Supplementary figures and images for: Tax abuse—The potential for the Sustainable Development Goals
Source: PLOS Glob Public Health. 2022 Feb 22;2(2):e0000119. doi: 10.1371/journal.pgph.0000119 (PMC10021515; doi:10.1371/journal.pgph.0000119)

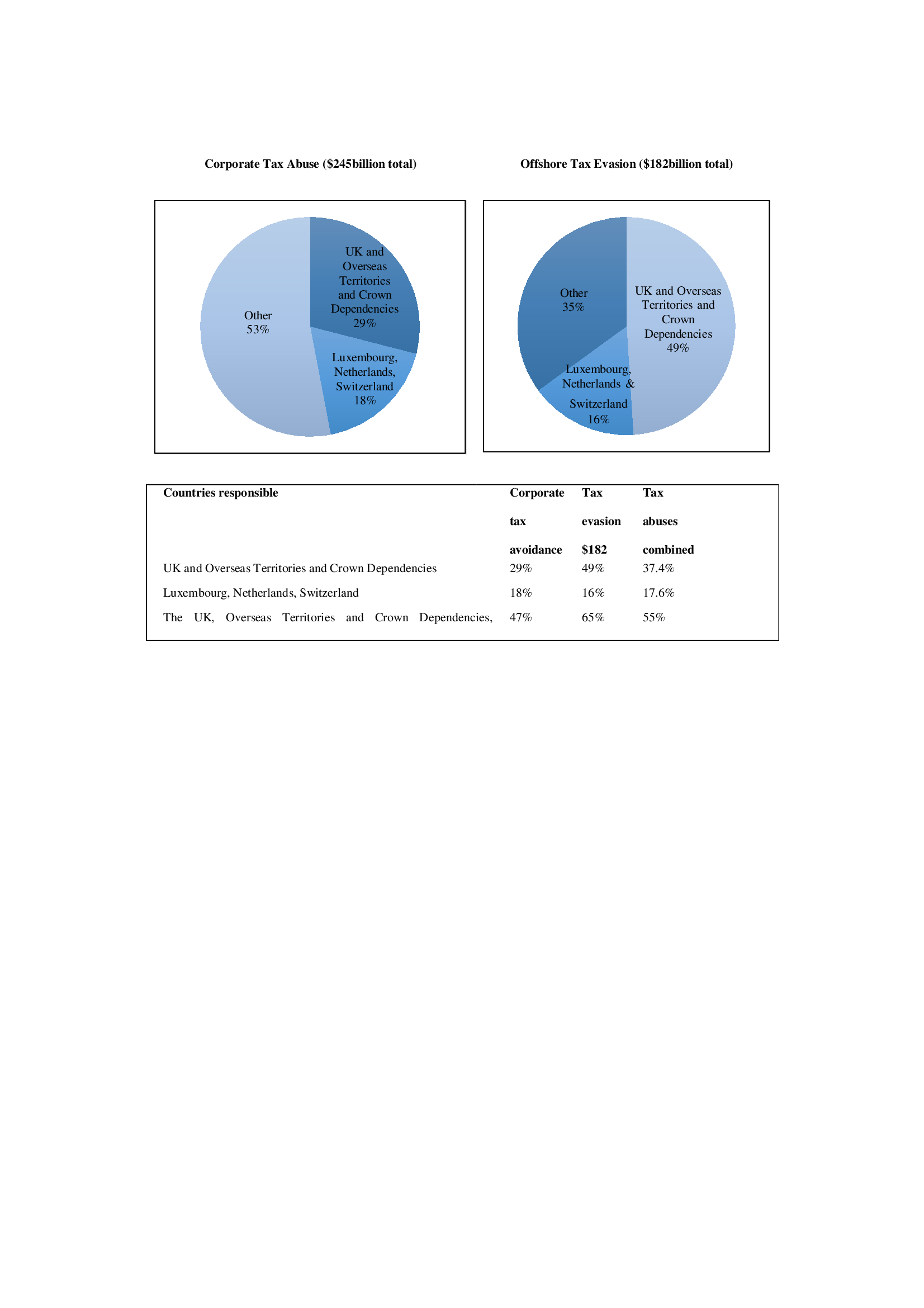

Supplement: S2 Fig — (TIFF) [file pgph.0000119.s002.tiff]
