# Supplementary material for: Tax abuse—The potential for the Sustainable Development Goals
Source: PLOS Glob Public Health. 2022 Feb 22;2(2):e0000119. doi: 10.1371/journal.pgph.0000119 (PMC10021515; doi:10.1371/journal.pgph.0000119)
Supplement: S1 Table — (DOCX) [file pgph.0000119.s003.docx]

| **Sustainable Development Goal** | **Summary** | **Targets** | **Indicators used in this study** |
| --- | --- | --- | --- |
| **SDG 3 -** **Good health and wellbeing** | Ensure healthy lives and promote well-being for all at all ages | -> By 2030, reduce the global maternal mortality ratio to less than 70 per 100,000 live births  -> By 2030, end preventable deaths of newborns and children under 5 years of age, with all countries aiming to reduce neonatal mortality to at least as low as 12 per 1,000 live births and under-5 mortality to at least as low as 25 per 1,000 live births | *Child and maternal mortality rates* |
| **SDG 4 – Quality education** | Ensure inclusive and equitable quality education and promote lifelong learning opportunities for all | -> By 2030, ensure that all girls and boys complete free, equitable and quality primary and secondary education leading to relevant and effective learning outcomes | *Additional school years* |
| **SDG 5 – Gender equality** | Achieve gender equality and empower all women and girls | -> End all forms of discrimination against all women and girls everywhere  -> Ensure universal access to sexual and reproductive health and reproductive rights | *Maternal mortality rates and access to drinking water/sanitation for women (see SDG 6)* |
| **SDG 6 – Clean water and sanitation** | Ensure availability and sustainable management of water and sanitation for all | -> By 2030, achieve universal and equitable access to safe and affordable drinking water for all  -> By 2030, achieve access to adequate and equitable sanitation and hygiene for all and end open defecation, paying special attention to the needs of women and girls and those in vulnerable situations | *Access to drinking water and sanitation* |
| *See S3 Table for definitions* | | | |
