# Supplementary material for: Tax abuse—The potential for the Sustainable Development Goals
Source: PLOS Glob Public Health. 2022 Feb 22;2(2):e0000119. doi: 10.1371/journal.pgph.0000119 (PMC10021515; doi:10.1371/journal.pgph.0000119)
Supplement: S2 Table — (DOCX) [file pgph.0000119.s004.docx]

| **Tax abuse** | In this paper, the term includes both tax avoidance and tax evasion. |
| --- | --- |
| **Tax Avoidance** | Legally reducing a tax bill using an unintended interpretation of tax codes by taking advantages of loopholes. Tax avoiders seek to comply with the letter of the law, but to subvert its purpose. |
| **Tax Haven or Low Tax Jurisdiction** | A country with low tax rates, either corporate or personal. Such places attract people to live or register companies to avoid paying a higher tax rate in their own country. Tax havens allow corporations and individuals to be less transparent by manipulating tax rules. Corporate tax abuse is facilitated by tax havens because they allow multinational corporations to shift profit which artificially drives down the amount they declare in the country where the profit was generated and artificially decrease the amount of tax due.  To facilitate tax evasion, tax havens pass laws to ensure secrecy and lack of transparency, and therefore they are sometimes called secrecy jurisdictions. |
| **Tax Evasion** | Illegal activity and the deliberate avoiding of tax. |
| **Gross National Income (GNI)** | the amount that a nations people and businesses earn. Our definitions are based country income levels from the World Banks Atlas method, most recently updated in 2019.  Low-income countries (LICs) have a GNI per capita of $1,035 or less. Lower-middle-income countries (LMICs) have a GNI per capita between $1,036 and $4,045. Upper-middle-income countries (UMICs) have a GNI per capita between $4046 and $12, 535. High-income countries (HICs) have a GNI per capita of $12536 or greater in 2019 |
