# Supplementary material for: Tax abuse—The potential for the Sustainable Development Goals
Source: PLOS Glob Public Health. 2022 Feb 22;2(2):e0000119. doi: 10.1371/journal.pgph.0000119 (PMC10021515; doi:10.1371/journal.pgph.0000119)
Supplement: S3 Table — (DOCX) [file pgph.0000119.s005.docx]

| **Basic drinking water services** | The percentage of the population drinking water from an improved source, provided collection time is not more than 30 minutes for a round trip. Improved water sources include piped water, boreholes, or tube wells, protected dug wells, protected springs, and packaged or delivered water. |
| --- | --- |
| **Safely managed drinking water services** | The percentage of the population using drinking water from an improved source accessible on-premises, available when needed and free from faecal and priority chemical contamination. |
| **Basic sanitation services** | The population using at least, that is, improved sanitation facilities not shared with other households. This indicator encompasses both people using basic sanitation services as well as those using safely managed sanitation services. Improved sanitation facilities include flush/pour flush to piped sewer systems, septic tanks, or pit latrines, ventilated improved pit latrines, compositing toilets, or pit latrines with slabs. |
| **Safely managed sanitation services** | The population using improved sanitation facilities, not shared with other households and where excreta are safely disposed of in situ or transported and treated offsite. Improved sanitation facilities include flush/pour flush to piped sewer systems, septic tanks, or pit latrines: ventilated improved pit latrines, compositing toilets, or pit latrines with slabs. |
| **School life expectancy (primary and secondary), both sexes (years)** | The number of years a person of school entrance age can expect to spend within the specified education level. For a child of a certain age, the school life expectancy is calculated as the sum of the age-specific enrolment rates for the levels of education specified. The part of the enrolment not distributed by age is divided by the school-age population for the level of education they are enrolled in, multiplied by the duration of that level of education. The result is added to the sum of the age-specific enrolment rates. A relatively high school life expectancy indicates a greater probability for children to spend more years in education and higher overall retention within the education system. Note that the expected number of years does not necessarily coincide with the expected number of education grades completed because of repetition. Since school life expectancy is an average based on participation in different levels of education, the expected number of years of schooling may be pulled down by the magnitude of children who never go to school. The GRADE uses the percentage of the maximum school life expectancy, both primary and secondary, for both sexes, globally, which is 17 years. |
