# Supplementary material for: Tax abuse—The potential for the Sustainable Development Goals
Source: PLOS Glob Public Health. 2022 Feb 22;2(2):e0000119. doi: 10.1371/journal.pgph.0000119 (PMC10021515; doi:10.1371/journal.pgph.0000119)
Supplement: S4 Table — (DOCX) [file pgph.0000119.s006.docx]

|  | **Additional numbers accessing basic drinking water** | | | **Additional numbers accessing safe drinking water** | | | **Additional numbers accessing basic sanitation** | | | **Additional numbers accessing safe sanitation** | | | **Number attending school for an extra year** | **Child deaths averted** | **Maternal deaths averted** |
| --- | --- | --- | --- | --- | --- | --- | --- | --- | --- | --- | --- | --- | --- | --- | --- |
|  | All | U5 | Women | All | U5 | Women | All | U5 | Women | All | U5 | Women |  |  |  |
| LIC | 1,726,659 | 296,849 | 406,722 | 97,548 | 16,764 | 23,058 | 2,918,981 | 523,418 | 675,862 | 17,253 | 2,969 | 4,068 | 187,989 | 116,180 | 14,840 |
| LMIC | 15,812,934 | 1,836,856 | 4,005,217 | 3,908,121 | 536,608 | 967,841 | 31,928,573 | 3,855,517 | 8,028,490 | 259,361 | 36,472 | 63,181 | 848,670 | 429,615 | 61,373 |
| UMIC | 523,123 | 49,259 | 139,131 | 3,684,316 | 310,248 | 992,064 | 833,375 | 82,672 | 221,023 | 1,253,722 | 96,198 | 340,466 | 521,510 | 59,827 | 1,512 |
| HIC | 3,977 | 398 | 1,037 | 693 | 38 | 177 | 17,471 | 1,482 | 4,395 | 1,999,205 | 122,816 | 472,592 | 5,283,985 | 6,061 | 26 |
| **Total** | **18,066,693** | **2,183,362** | **4,552,107** | **7,690,678** | **863,658** | **1,983,140** | **35,698,400** | **4,463,089** | **8,929,770** | **3,529,541** | **258,455** | **880,307** | **6,842,154** | **611,683** | **77,751** |
| ***Sources: UNU-Wider, 2020; World Bank, 2018; GRADE, 2021*** | | | | | | | | | | | | | | | |
