# Supplementary material for: Tax abuse—The potential for the Sustainable Development Goals
Source: PLOS Glob Public Health. 2022 Feb 22;2(2):e0000119. doi: 10.1371/journal.pgph.0000119 (PMC10021515; doi:10.1371/journal.pgph.0000119)
Supplement: S5 Table — (DOCX) [file pgph.0000119.s007.docx]

| **The UK and Overseas Territories and Crown Dependencies, Luxembourg, Netherlands, Switzerland** | **Additional numbers accessing basic drinking water** | | | **Additional numbers accessing safe drinking water** | | | **Additional numbers accessing basic sanitation** | | | **Additional numbers accessing safe sanitation** | | | **Number attending school for an extra year** | **Child deaths averted** | **Maternal deaths averted** |
| --- | --- | --- | --- | --- | --- | --- | --- | --- | --- | --- | --- | --- | --- | --- | --- |
|  | All | U5 | Women | All | U5 | Women | All | U5 | Women | All | U5 | Women |  |  |  |
|  | 9,936,681 | 1,200,849 | 2,503,659 | 4,229,873 | 475,012 | 1,090,727 | 19,634,120 | 2,454,699 | 4,911,374 | 1,941,248 | 142,150 | 484,169 | 3,763,185 | 336,426 | 42,763 |
| ***Sources: UNU-Wider, 2020; World Bank, 2018; GRADE, 2021*** | | | | | | | | | | | | | | | |
