# Supplementary material for: Tax abuse—The potential for the Sustainable Development Goals
Source: PLOS Glob Public Health. 2022 Feb 22;2(2):e0000119. doi: 10.1371/journal.pgph.0000119 (PMC10021515; doi:10.1371/journal.pgph.0000119)
Supplement: S6 Table — (DOCX) [file pgph.0000119.s008.docx]

| **The UK and Overseas Territories and Crown Dependencies** | **Additional numbers accessing basic drinking water** | | | **Additional numbers accessing safe drinking water** | | | **Additional numbers accessing basic sanitation** | | | **Additional numbers accessing safe sanitation** | | | **Number attending school for an extra year** | **Child deaths averted** | **Maternal deaths averted** |
| --- | --- | --- | --- | --- | --- | --- | --- | --- | --- | --- | --- | --- | --- | --- | --- |
|  | All | U5 | Women | All | U5 | Women | All | U5 | Women | All | U5 | Women |  |  |  |
|  | 6,759,623 | 816,901 | 1,703,163 | 2,877,454 | 323,136 | 741,989 | 13,356,497 | 1,669,857 | 3,341,058 | 1,320,572 | 96,701 | 329,365 | 2,559,980 | 228,860 | 29,090 |
| ***Sources: UNU-Wider, 2020; World Bank, 2018; GRADE, 2021*** | | | | | | | | | | | | | | | |
