# Supplementary material for: Tax abuse—The potential for the Sustainable Development Goals
Source: PLOS Glob Public Health. 2022 Feb 22;2(2):e0000119. doi: 10.1371/journal.pgph.0000119 (PMC10021515; doi:10.1371/journal.pgph.0000119)
Supplement: S7 Table — (DOCX) [file pgph.0000119.s009.docx]

| Country | Tax loss constant 2010 USD | Additional numbers accessing basic drinking water | | | Additional numbers accessing safe drinking water | | | Additional numbers accessing basic sanitation | | | Additional numbers accessing safe sanitation | | | Number attending school for an extra year | Child deaths averted | Maternal deaths averted |
| --- | --- | --- | --- | --- | --- | --- | --- | --- | --- | --- | --- | --- | --- | --- | --- | --- |
|  |  | All | U5 | Women | All | U5 | Women | All | U5 | Women | All | U5 | Women |  |  |  |
| Afghanistan | 2,539,977.07 | 832 | 136 | 188 | n/a | n/a | n/a | 5,239 | 886 | 1,160 | n/a | n/a | n/a | 490 | 243 | 52 |
| Benin | 2,193,245.80 | 1,363 | 227 | 319 | n/a | n/a | n/a | 1,254 | 209 | 293 | n/a | n/a | n/a | n/a | 41 | 6 |
| Burkina Faso | 2,516,825.17 | 2,364 | 416 | 540 | n/a | n/a | n/a | 2,651 | 467 | 605 | n/a | n/a | n/a | 213 | 74 | 13 |
| Burundi | 1,731,461.79 | n/a | n/a | n/a | n/a | n/a | n/a | n/a | n/a | n/a | n/a | n/a | n/a | n/a | n/a | n/a |
| Central African Republic | 33,364,659.42 | n/a | n/a | n/a | n/a | n/a | n/a | n/a | n/a | n/a | n/a | n/a | n/a | n/a | n/a | n/a |
| Chad | 316,587,778.00 | 115,407 | 21,906 | 25,078 | n/a | n/a | n/a | 715,816 | 136,806 | 155,076 | n/a | n/a | n/a | 66,898 | 34,733 | 3,580 |
| Comoros | 283,933.81 | 34 | 5 | 8 | n/a | n/a | n/a | 27 | 4 | 7 | n/a | n/a | n/a | 14 | 9 | 1 |
| Congo DRC | 104,138,977.10 | 5,841 | 1,082 | 1,293 | n/a | n/a | n/a | 141,440 | 26,314 | 31,419 | n/a | n/a | n/a | n/a | 12,117 | 1,681 |
| Eritrea | 1,249,354.06 | n/a | n/a | n/a | n/a | n/a | n/a | n/a | n/a | n/a | n/a | n/a | n/a | n/a | n/a | n/a |
| Ethiopia | 344,421,430.50 | n/a | n/a | n/a | n/a | n/a | n/a | n/a | n/a | n/a | n/a | n/a | n/a | n/a | n/a | n/a |
| Gambia | 180,388,457.90 | 138,553 | 24,547 | 33,356 | n/a | n/a | n/a | 230,762 | 49,875 | 55,568 | n/a | n/a | n/a | n/a | 4,756 | 581 |
| Guinea | 3,438,436.14 | 1,258 | 214 | 301 | n/a | n/a | n/a | 2,968 | 506 | 709 | n/a | n/a | n/a | n/a | 134 | 25 |
| Guinea-Bissau | 15,776,437.85 | 2,283 | 383 | 561 | n/a | n/a | n/a | 9,331 | 1,552 | 2,311 | n/a | n/a | n/a | n/a | 605 | 71 |
| Haiti | 76,413,964.41 | 2,002 | 283 | 536 | n/a | n/a | n/a | 34,754 | 4,288 | 9,114 | n/a | n/a | n/a | n/a | 2,528 | 195 |
| Liberia | 168,955,073.30 | 131,771 | 20,992 | 30,988 | n/a | n/a | n/a | 129,505 | 20,570 | 30,465 | n/a | n/a | n/a | n/a | 13,455 | 1,679 |
| Madagascar | 68,301,278.16 | 26,332 | 4,141 | 6,261 | n/a | n/a | n/a | 34,852 | 5,460 | 8,294 | n/a | n/a | n/a | 3,352 | 1,455 | 189 |
| Malawi | 51,310,721.97 | 64,220 | 11,115 | 14,995 | n/a | n/a | n/a | 52,826 | 9,132 | 12,332 | n/a | n/a | n/a | 4,688 | 1,653 | 208 |
| Mali | 13,293,901.13 | 5,930 | 1,123 | 1,303 | n/a | n/a | n/a | 8,193 | 1,548 | 1,801 | 265 | 50 | 58 | 876 | 340 | 52 |
| Mozambique | 433,321,234.00 | 572,388 | 99,140 | 134,577 | n/a | n/a | n/a | 724,449 | 124,914 | 170,450 | n/a | n/a | n/a | 63,222 | 22,879 | 3,671 |
| Nepal | 8,068,794.09 | 5,698 | 606 | 1,606 | 5,546 | 589 | 1,563 | 7,676 | 820 | 2,149 | n/a | n/a | n/a | 450 | 166 | 26 |
| Niger | 11,926,373.54 | 10,568 | 2,166 | 2,205 | n/a | n/a | n/a | 13,542 | 2,777 | 2,827 | 384 | 79 | 80 | 1,344 | 482 | 84 |
| North Korea | 473,332,422.30 | n/a | n/a | n/a | n/a | n/a | n/a | n/a | n/a | n/a | n/a | n/a | n/a | n/a | n/a | n/a |
| Rwanda | 65,391,812.74 | 100,779 | 15,190 | 25,238 | n/a | n/a | n/a | 91,438 | 13,823 | 22,920 | n/a | n/a | n/a | 3,737 | 1,379 | 276 |
| Senegal | 151,938,765.70 | 105,041 | 17,725 | 25,827 | n/a | n/a | n/a | 111,569 | 18,869 | 27,429 | 3,749 | 635 | 922 | 8,173 | 2,447 | 343 |
| Sierra Leone | 69,456,955.26 | 31,263 | 4,934 | 7,434 | 35,364 | 5,563 | 8,416 | 36,374 | 5,767 | 8,633 | 1,417 | 225 | 336 | 5,005 | 2,560 | 284 |
| Somalia | 254,141.90 | n/a | n/a | n/a | n/a | n/a | n/a | n/a | n/a | n/a | n/a | n/a | n/a | n/a | n/a | n/a |
| South Sudan | 6,584,940.48 | n/a | n/a | n/a | n/a | n/a | n/a | n/a | n/a | n/a | n/a | n/a | n/a | n/a | n/a | n/a |
| Syria | 8,109,481.72 | n/a | n/a | n/a | n/a | n/a | n/a | n/a | n/a | n/a | n/a | n/a | n/a | n/a | n/a | n/a |
| Tajikistan | 171,726,346.60 | n/a | n/a | n/a | n/a | n/a | n/a | n/a | n/a | n/a | n/a | n/a | n/a | n/a | n/a | n/a |
| Tanzania | 271,486,855.70 | 285,547 | 49,378 | 66,661 | n/a | n/a | n/a | 323,537 | 55,870 | 75,558 | 11,438 | 1,980 | 2,672 | 25,414 | 8,607 | 1,154 |
| Togo | 37,728,766.51 | 14,088 | 2,239 | 3,409 | n/a | n/a | n/a | 21,868 | 3,494 | 5,286 | n/a | n/a | n/a | 2,309 | 1,120 | 112 |
| Uganda | 104,162,490.10 | 89,100 | 16,680 | 20,583 | 56,638 | 10,612 | 13,079 | 168,263 | 31,484 | 38,876 | n/a | n/a | n/a | n/a | 2,423 | 297 |
| Yemen | 50,463,820.14 | 8716 | 1369 | 2087 | n/a | n/a | n/a | 31150 | 4859 | 7509 | n/a | n/a | n/a | 1804 | 659 | 81 |
| Zimbabwe | 107,725,783.30 | n/a | n/a | n/a | n/a | n/a | n/a | n/a | n/a | n/a | n/a | n/a | n/a | n/a | n/a | n/a |
| Total | | **1,726,659** | **296,849** | **406,722** | **97,548** | **16,764** | **23,058** | **2,918,981** | **523,418** | **675,862** | **17,253** | **2,969** | **4,068** | **187,989** | **116,180** | **14,840** |
