# Supplementary material for: Tax abuse—The potential for the Sustainable Development Goals
Source: PLOS Glob Public Health. 2022 Feb 22;2(2):e0000119. doi: 10.1371/journal.pgph.0000119 (PMC10021515; doi:10.1371/journal.pgph.0000119)
Supplement: S8 Table — (DOCX) [file pgph.0000119.s010.docx]

| Country | Tax loss constant 2010 USD | Additional numbers accessing basic drinking water | | | Additional numbers accessing safe drinking water | | | Additional numbers accessing basic sanitation | | | Additional numbers accessing safe sanitation | | | Number attending school for an extra year | Child deaths averted | Maternal deaths averted |
| --- | --- | --- | --- | --- | --- | --- | --- | --- | --- | --- | --- | --- | --- | --- | --- | --- |
|  |  | All | U5 | Women | All | U5 | Women | All | U5 | Women | All | U5 | Women |  |  |  |
| Angola | 2,040,831,959.00 | 584,200 | 110,120 | 132,686 | n/a | n/a | n/a | 1,121,622 | 210,377 | 254,755 | n/a | n/a | n/a | n/a | 44,273 | 2,529 |
| Bangladesh | 638,345,219.30 | 435,377 | 42,580 | 120,244 | 250,840 | 24,169 | 69,530 | 645,183 | 63,146 | 178,073 | n/a | n/a | n/a | 29,079 | 9,805 | 1,677 |
| Bhutan | 77,394.55 | 83 | 8 | 22 | 180 | 16 | 47 | 21 | 2 | 6 | n/a | n/a | n/a | 2 | 1 | - |
| Bolivia | 122,179,971.80 | 55,928 | 6,379 | 13,963 | n/a | n/a | n/a | 96,540 | 10,995 | 24,114 | 1,915 | 218 | 479 | 7,097 | 1,730 | 282 |
| Cambodia | 21,147,950.64 | 18,665 | 2,124 | 5,087 | 9,609 | 1,101 | 2,622 | 44,203 | 5,034 | 12,051 | n/a | n/a | n/a | n/a | 511 | 63 |
| Cameroon | 126,611,667.80 | 48,333 | 8,079 | 11,495 | n/a | n/a | n/a | 111,354 | 18,594 | 26,480 | n/a | n/a | n/a | n/a | 2,370 | 303 |
| Cape Verde | 1,077,905.85 | 552 | 58 | 147 | n/a | n/a | n/a | 380 | 40 | 101 | n/a | n/a | n/a | 36 | 8 | 1 |
| Congo, Rep. | 11,328,439.60 | 2,781 | 453 | 680 | 4,923 | 803 | 1,206 | 6,725 | 1,093 | 1,644 | n/a | n/a | n/a | n/a | 200 | 13 |
| Cote d'Ivoire | 215,446,635.80 | 86,978 | 13,916 | 20,464 | 106,237 | 16,991 | 24,953 | 160,253 | 25,661 | 37,474 | n/a | n/a | n/a | 12,851 | 4,875 | 741 |
| Djibouti | 3,872,419.75 | 1,128 | 123 | 293 | n/a | n/a | n/a | 1,883 | 205 | 490 | n/a | n/a | n/a | 86 | 36 | 4 |
| Egypt | 2,102,225,005.00 | n/a | n/a | n/a | n/a | n/a | n/a | n/a | n/a | n/a | n/a | n/a | n/a | n/a | n/a | n/a |
| El Salvador | 97,292,394.20 | 36,586 | 3,486 | 10,123 | n/a | n/a | n/a | 65,996 | 6,274 | 18,273 | n/a | n/a | n/a | 2,437 | 529 | 80 |
| Eswatini | 16,049,051.69 | 3,281 | 449 | 879 | n/a | n/a | n/a | 6,279 | 861 | 1,684 | n/a | n/a | n/a | n/a | 167 | 13 |
| Georgia | 66,941,870.29 | n/a | n/a | n/a | n/a | n/a | n/a | n/a | n/a | n/a | n/a | n/a | n/a | n/a | n/a | n/a |
| Ghana | 140,788,905.20 | 214,592 | 30,870 | 53,608 | 144,867 | 20,829 | 36,192 | 206,091 | 29,625 | 51,477 | n/a | n/a | n/a | 10,940 | 2,863 | 412 |
| Honduras | 299,094,116.40 | 115,456 | 13,413 | 30,361 | n/a | n/a | n/a | 250,305 | 29,131 | 65,787 | n/a | n/a | n/a | 11,674 | 3,069 | 370 |
| India | 9,373,795,704.00 | 7,214,298 | 697,745 | 1,847,320 | n/a | n/a | n/a | 12,400,238 | 1,196,234 | 3,176,544 | n/a | n/a | n/a | 378,242 | 81,479 | 13,486 |
| Indonesia | 4,419,505,046.00 | 1,736,659 | 163,922 | 470,567 | n/a | n/a | n/a | 3,816,719 | 360,249 | 1,034,747 | n/a | n/a | n/a | 128,194 | 27,496 | 3,858 |
| Kenya | 511,997,825.00 | 284,178 | 43,127 | 71,374 | n/a | n/a | n/a | 631,109 | 96,103 | 158,387 | n/a | n/a | n/a | n/a | 7,049 | 1,239 |
| Kiribati | 170,640.41 | 45 | 6 | 12 | n/a | n/a | n/a | -6 | -1 | -2 | n/a | n/a | n/a | n/a | 2 | - |
| Kyrgyz Republic | 14,771,114.56 | 7,279 | 893 | 1,954 | 594 | 80 | 150 | 18,986 | 2,314 | 5,110 | n/a | n/a | n/a | 805 | 231 | 31 |
| Laos | 78,851,783.58 | 73,471 | 8,783 | 19,620 | 85,302 | 10,231 | 22,780 | 138,557 | 16,630 | 36,985 | 4,435 | 531 | 1,184 | 4,081 | 1,729 | 217 |
| Lesotho | 253,729,969.20 | 91,815 | 11,227 | 23,954 | n/a | n/a | n/a | 100,539 | 12,310 | 26,232 | n/a | n/a | n/a | 9,205 | 2,712 | 271 |
| Mauritania | 16,784,372.67 | 5,573 | 872 | 1,356 | n/a | n/a | n/a | 11,437 | 1,789 | 2,784 | n/a | n/a | n/a | 780 | 274 | 31 |
| Micronesia | 233,548.28 | 79 | 9 | 20 | n/a | n/a | n/a | -25 | -3 | -6 | n/a | n/a | n/a | n/a | 2 | - |
| Moldova | 26,625,677.50 | 7,900 | 423 | 2,127 | 11,478 | 612 | 3,099 | 16,329 | 874 | 4,399 | n/a | n/a | n/a | 442 | 91 | 7 |
| Mongolia | 38,374,567.57 | 15,115 | 1,701 | 4,398 | 24,379 | 2,784 | 7,066 | 32,880 | 3,730 | 9,535 | n/a | n/a | n/a | n/a | 425 | 29 |
| Morocco | 471,481,137.50 | 127,749 | 12,620 | 34,555 | 431,890 | 42,692 | 116,660 | 233,240 | 23,043 | 62,919 | 19,363 | 1,913 | 5,250 | 14,248 | 3,056 | 242 |
| Myanmar | 3,551,236.06 | 4,414 | 387 | 1,234 | n/a | n/a | n/a | 10,803 | 979 | 3,019 | n/a | n/a | n/a | 668 | 337 | 37 |
| Nicaragua | 71,451,874.84 | n/a | n/a | n/a | n/a | n/a | n/a | n/a | n/a | n/a | n/a | n/a | n/a | n/a | n/a | n/a |
| Nigeria | 9,832,106,123.00 | 1,940,257 | 338,523 | 443,378 | 1,410,968 | 246,764 | 323,128 | 5,508,715 | 963,018 | 1,259,056 | 134,478 | 23,513 | 30,736 | n/a | 151,877 | 21,446 |
| Pakistan | 2,301,064,322.00 | 1005979 | 131759 | 245610 | 844133 | 110175 | 206140 | 2655471 | 348229 | 648283 | n/a | n/a | n/a | 104698 | 32557 | 6848 |
| Papua New Guinea | 19,083,840.53 | 9,820 | 1,315 | 2,432 | - | - | - | 18,989 | 2,549 | 4,701 | n/a | n/a | n/a | n/a | 302 | 39 |
| Philippines | 1,931,441,527.00 | 886,289 | 101,773 | 228,445 | 327,468 | 36,772 | 84,424 | 1,921,454 | 220,583 | 495,210 | 78,539 | 9,006 | 20,232 | 73,559 | 14,212 | 3,169 |
| Sao Tome and Principe | 135,417.97 | 80 | 13 | 19 | n/a | n/a | n/a | 25 | 4 | 6 | n/a | n/a | n/a | 8 | 3 | 1 |
| Solomon Islands | 1,770,475.48 | 1,547 | 237 | 377 | n/a | n/a | n/a | 449 | 69 | 109 | n/a | n/a | n/a | n/a | 73 | 7 |
| Sri Lanka | 94,998,760.91 | 55,512 | 4,735 | 14,458 | n/a | n/a | n/a | 95,374 | 8,157 | 24,878 | n/a | n/a | n/a | 2,442 | 569 | 67 |
| Sudan | 586,347,896.50 | 187004 | 29671 | 44090 | n/a | n/a | n/a | 787112 | 124536 | 185725 | n/a | n/a | n/a | 51496 | 24149 | 2757 |
| Timor-Leste | 614,827.85 | 893 | 124 | 205 | n/a | n/a | n/a | 622 | 87 | 142 | n/a | n/a | n/a | 177 | 70 | 11 |
| Tunisia | 267,829,120.40 | 15,090 | 1,288 | 4,186 | 128,968 | 11,193 | 35,694 | 62,028 | 5,562 | 17,084 | 6,432 | 538 | 1,788 | n/a | 1,095 | 55 |
| Ukraine | 589,875,038.10 | 75,515 | 3,978 | 18,798 | 39,131 | 2,006 | 9,795 | 183,019 | 9,619 | 45,536 | 14,199 | 753 | 3,512 | n/a | 1,515 | 81 |
| Uzbekistan | 159,110,475.70 | 67,486 | 7,106 | 18,988 | 81,588 | 8,593 | 22,965 | 68,924 | 7,084 | 19,584 | n/a | n/a | n/a | 5,423 | 2,010 | 237 |
| Vanuatu | 4,690,100.33 | 3,064 | 440 | 766 | 5,566 | 797 | 1,390 | 1,485 | 212 | 370 | n/a | n/a | n/a | n/a | 66 | 6 |
| Vietnam | 380,543,397.50 | 287,608 | 23,595 | 80,304 | n/a | n/a | n/a | 395,007 | 32,420 | 110,577 | n/a | n/a | n/a | n/a | 2,638 | 350 |
| Zambia | 128,922,807.10 | 104,275 | 18,526 | 24,618 | n/a | n/a | n/a | 102,258 | 18,099 | 24,167 | n/a | n/a | n/a | n/a | 3,159 | 363 |
| Total | | **15,812,934** | **1,836,856** | **4,005,217** | **3,908,121** | **536,608** | **967,841** | **31,928,573** | **3,855,517** | **8,028,490** | **259,361** | **36,472** | **63,181** | **848,670** | **429,615** | **61,373** |
