# Supplementary material for: Tax abuse—The potential for the Sustainable Development Goals
Source: PLOS Glob Public Health. 2022 Feb 22;2(2):e0000119. doi: 10.1371/journal.pgph.0000119 (PMC10021515; doi:10.1371/journal.pgph.0000119)
Supplement: S9 Table — (DOCX) [file pgph.0000119.s011.docx]

| Country | Tax loss constant 2010 USD | Additional numbers accessing basic drinking water | | | Additional numbers accessing safe drinking water | | | Additional numbers accessing basic sanitation | | | Additional numbers accessing safe sanitation | | | Number attending school for an extra year | Child deaths averted | Maternal deaths averted | |
| --- | --- | --- | --- | --- | --- | --- | --- | --- | --- | --- | --- | --- | --- | --- | --- | --- | --- |
|  |  | **All** | **U5** | **Women** | **All** | **U5** | **Women** | **All** | **U5** | **Women** | **All** | **U5** | **Women** |  |  |  |  |
| Albania | 42,826,051.19 | 6,567 | 392 | 1,638 | 9,754 | 583 | 2,412 | 11,386 | 682 | 2,840 | 1,989 | 119 | 492 | 704 | 116 | 6 |  |
| Algeria | 445,753,666.40 | 25,528 | 2,898 | 7,004 | n/a | n/a | n/a | 101,132 | 11,536 | 27,761 | 1,416 | 149 | 397 | n/a | 2,222 | 90 |  |
| Armenia | 30,559,851.03 | 8,315 | 591 | 2,295 | 22,093 | 1,588 | 6,017 | 6,543 | 469 | 1,790 | 2,553 | 183 | 697 | 802 | 155 | 11 |  |
| Azerbaijan | 29,365,000.42 | 2,523 | 207 | 735 | 15,138 | 1,272 | 4,349 | 229 | 19 | 67 | n/a | n/a | n/a | 1,856 | 224 | 7 |  |
| Belarus | 60,616,913.71 | 3,663 | 203 | 936 | 2,161 | 124 | 540 | 7,870 | 445 | 1,987 | 634 | 37 | 158 | 928 | 140 | 4 |  |
| Belize | 97,671,516.61 | 10,538 | 1,186 | 2,843 | n/a | n/a | n/a | 32,145 | 3,619 | 8,670 | n/a | n/a | n/a | 3,509 | 611 | 30 |  |
| Bosnia and Herzegovina | 17,239,226.50 | 394 | 19 | 97 | 2,187 | 103 | 532 | 1,093 | 52 | 269 | 508 | 24 | 122 | n/a | 24 | 1 |  |
| Botswana | 21,254,981.78 | 17 | 2 | 5 | n/a | n/a | n/a | 855 | 108 | 234 | n/a | n/a | n/a | n/a | 54 | - |  |
| Brazil | 13,544,758,426.00 | 478 | 36 | 133 | n/a | n/a | n/a | 11,665 | 906 | 3,261 | 385,951 | 28,565 | 106,670 | n/a | 8,218 | 7 |  |
| Bulgaria | 37,234,012.64 | 71 | 4 | 16 | 5,003 | 244 | 1,121 | 109 | 5 | 25 | 1,682 | 81 | 375 | 379 | 28 | - |  |
| China | 13,112,491,905.00 | n/a | n/a | n/a | n/a | n/a | n/a | n/a | n/a | n/a | n/a | n/a | n/a | n/a | n/a | n/a |  |
| Colombia | 10,699,220,288.00 | 97,198 | 8,003 | 26,658 | 2,469,383 | 200,822 | 676,561 | 78,850 | 6,542 | 21,645 | 562,435 | 45,349 | 153,952 | 268,344 | 22,714 | 383 |  |
| Costa Rica | 189,628,612.40 | 786 | 60 | 213 | 15,917 | 1,217 | 4,312 | 21,822 | 1,661 | 5,907 | n/a | n/a | n/a | 3,509 | 352 | 5 |  |
| Cuba | 1,247,946.11 | n/a | n/a | n/a | n/a | n/a | n/a | n/a | n/a | n/a | n/a | n/a | n/a | n/a | n/a | n/a |  |
| Dominica | 4,186,899.88 | n/a | n/a | n/a | n/a | n/a | n/a | n/a | n/a | n/a | n/a | n/a | n/a | n/a | n/a | n/a |  |
| Dominican Republic | 110,809,123.10 | 34,476 | 3,462 | 9,107 | n/a | n/a | n/a | 73,312 | 7,360 | 19,358 | n/a | n/a | n/a | 3,885 | 788 | 55 |  |
| Ecuador | 273,353,902.20 | n/a | n/a | n/a | n/a | n/a | n/a | n/a | n/a | n/a | n/a | n/a | n/a | n/a | n/a | n/a |  |
| Equatorial Guinea | 3,475,802.09 | 208 | 32 | 46 | n/a | n/a | n/a | 77 | 11 | 17 | n/a | n/a | n/a | n/a | 18 | 2 |  |
| Fiji | 3,767,089.72 | 725 | 77 | 187 | n/a | n/a | n/a | 1,259 | 134 | 324 | n/a | n/a | n/a | n/a | 23 | 2 |  |
| Gabon | 140,156,173.90 | 4,611 | 672 | 1,163 | n/a | n/a | n/a | 7,811 | 1,141 | 1,977 | n/a | n/a | n/a | n/a | 592 | 34 |  |
| Grenada | 2,660,992.48 | 74 | 6 | 19 | 587 | 49 | 150 | 584 | 49 | 149 | n/a | n/a | n/a | 44 | 6 | - |  |
| Guatemala | 33,120,148.30 | 10631 | 1379 | 2723 | 5777 | 737 | 1481 | 21579 | 2799 | 5529 | n/a | n/a | n/a | 1131 | 319 | 65 |  |
| Guyana | 261,331,280.20 | n/a | n/a | n/a | n/a | n/a | n/a | n/a | n/a | n/a | n/a | n/a | n/a | n/a | n/a | n/a |  |
| Iran | 8,401,235.03 | n/a | n/a | n/a | n/a | n/a | n/a | n/a | n/a | n/a | n/a | n/a | n/a | n/a | n/a | n/a |  |
| Iraq | 5,635,250.30 | n/a | n/a | n/a | n/a | n/a | n/a | n/a | n/a | n/a | n/a | n/a | n/a | n/a | n/a | n/a |  |
| Jamaica | 25,854,730.40 | n/a | n/a | n/a | n/a | n/a | n/a | n/a | n/a | n/a | n/a | n/a | n/a | n/a | n/a | n/a |  |
| Jordan | 129,708,544.50 | 31,278 | 4,046 | 7,985 | 107,000 | 13,827 | 27,320 | 40,685 | 5,209 | 10,403 | 4,932 | 647 | 1,256 | 4,212 | 888 | 75 |  |
| Kazakhstan | 237,027,446.50 | 5,319 | 538 | 1,446 | 49,068 | 5,076 | 13,138 | 1,549 | 152 | 430 | n/a | n/a | n/a | 6,310 | 808 | 27 |  |
| Lebanon | 126,553,426.50 | 9,543 | 840 | 2,620 | 16,709 | 1,405 | 4,575 | 9,195 | 827 | 2,525 | 5,123 | 432 | 1,406 | n/a | 435 | 19 |  |
| Libya | 47,676,673.78 | 126 | 15 | 39 | n/a | n/a | n/a | 550 | 57 | 158 | 461 | 47 | 134 | n/a | 54 | 2 |  |
| Malaysia | 1,103,376,488.00 | 2,211 | 191 | 597 | 136,800 | 11,885 | 36,855 | 639 | 55 | 173 | 31,610 | 2,704 | 8,564 | 15,860 | 2,294 | 29 |  |
| Maldives | 598,419.89 | 34 | 3 | 9 | n/a | n/a | n/a | 86 | 8 | 23 | n/a | n/a | n/a | 15 | 2 | - |  |
| Marshall Islands | 71,749,911.41 | n/a | n/a | n/a | n/a | n/a | n/a | n/a | n/a | n/a | n/a | n/a | n/a | n/a | n/a | n/a |  |
| Mauritius | 150,593,545.10 | 988 | 65 | 263 | n/a | n/a | n/a | 2,136 | 143 | 573 | n/a | n/a | n/a | 1,917 | 221 | 4 |  |
| Mexico | 8,212,263,152.00 | n/a | n/a | n/a | n/a | n/a | n/a | n/a | n/a | n/a | n/a | n/a | n/a | n/a | n/a | n/a |  |
| Montenegro | 97,325,180.03 | n/a | n/a | n/a | n/a | n/a | n/a | n/a | n/a | n/a | n/a | n/a | n/a | n/a | n/a | n/a |  |
| Namibia | 25,005,073.63 | 534 | 73 | 141 | n/a | n/a | n/a | 6,399 | 879 | 1,692 | n/a | n/a | n/a | n/a | 152 | 3 |  |
| Nauru | 970.40 | n/a | n/a | n/a | n/a | n/a | n/a | n/a | n/a | n/a | n/a | n/a | n/a | n/a | n/a | n/a |  |
| North Macedonia | 24,575,964.00 | 1,016 | 55 | 255 | 6,932 | 378 | 1,737 | 1,894 | 103 | 478 | 815 | 45 | 204 | 263 | 37 | 1 |  |
| Paraguay | 90,164,378.23 | 30,898 | 3,275 | 7,952 | 26,591 | 2,793 | 6,857 | 70,038 | 7,412 | 18,030 | 2,668 | 281 | 688 | n/a | 895 | 82 |  |
| Peru | 1,091,592,610.00 | 165,051 | 16,029 | 43,538 | 374,982 | 36,758 | 98,900 | 256,446 | 24,961 | 67,168 | 62,010 | 5,940 | 16,340 | 41,420 | 5,944 | 394 |  |
| Romania | 794,458,514.40 | n/a | n/a | n/a | n/a | n/a | n/a | n/a | n/a | n/a | n/a | n/a | n/a | n/a | n/a | n/a |  |
| Russia | 4,622,011,403.00 | 2,004 | 122 | 521 | 337,606 | 20,940 | 86,995 | 61 | 4 | 15 | 185,898 | 11,333 | 48,315 | 70,006 | 2,834 | 6 |  |
| Samoa | 140,844,191.00 | 8,355 | 1,192 | 1,883 | 68,332 | 9,811 | 15,352 | 3,890 | 558 | 874 | 1,150 | 165 | 258 | n/a | 607 | 26 |  |
| Serbia | 67,617,669.56 | 373 | 20 | 87 | 12,296 | 636 | 2,860 | 1,862 | 99 | 435 | 1,887 | 97 | 438 | 729 | 66 | 1 |  |
| South Africa | 3,057,752,265.00 | 1,658 | 177 | 459 | n/a | n/a | n/a | 15,122 | 1,606 | 4,186 | n/a | n/a | n/a | 81,082 | 5,819 | 28 |  |
| St. Lucia | 7,268,170.71 | 96 | 6 | 27 | n/a | n/a | n/a | 1,620 | 102 | 460 | n/a | n/a | n/a | 93 | 12 | - |  |
| St. Vincent & Grenadines | 24,801,186.79 | 208 | 17 | 53 | n/a | n/a | n/a | 4,065 | 324 | 1,040 | n/a | n/a | n/a | 342 | 44 | 1 |  |
| Suriname | 10,017,171.95 | 273 | 26 | 71 | n/a | n/a | n/a | 2,393 | 232 | 620 | n/a | n/a | n/a | 294 | 36 | 1 |  |
| Thailand | 1,031,452,469.00 | 55,951 | 3,287 | 15,269 | n/a | n/a | n/a | 33,142 | 1,981 | 9,136 | - | - | - | 13,876 | 2,030 | 107 |  |
| Tonga | 8,370,005.83 | 404 | 53 | 98 | n/a | n/a | n/a | 3,282 | 422 | 794 | n/a | n/a | n/a | n/a | 45 | 4 |  |
| Turkey | 2,425,226,219.00 | n/a | n/a | n/a | n/a | n/a | n/a | n/a | n/a | n/a | n/a | n/a | n/a | n/a | n/a | n/a |  |
| Turkmenistan | 426,055.40 | n/a | n/a | n/a | n/a | n/a | n/a | n/a | n/a | n/a | n/a | n/a | n/a | n/a | n/a | n/a |  |
| Venezuela | 574,129,475.50 | n/a | n/a | n/a | n/a | n/a | n/a | n/a | n/a | n/a | n/a | n/a | n/a | n/a | n/a | n/a |  |
| Total | | **523,123** | **49,259** | **139,131** | **3,684,316** | **310,248** | **992,064** | **833,375** | **82,672** | **221,023** | **1,253,722** | **96,198** | **340,466** | **521,510** | **59,827** | **1,512** |  |
