# Supplementary material for: Tax abuse—The potential for the Sustainable Development Goals
Source: PLOS Glob Public Health. 2022 Feb 22;2(2):e0000119. doi: 10.1371/journal.pgph.0000119 (PMC10021515; doi:10.1371/journal.pgph.0000119)
Supplement: S10 Table — (DOCX) [file pgph.0000119.s012.docx]

**S10 Table: Potential for progress towards the SDGs associated with increased government revenue equivalent to the tax abuse for high-income countries**

| **Country** | **Tax loss constant 2010 USD** | **Additional numbers accessing basic drinking water** | | | **Additional numbers accessing safe drinking water** | | | **Additional numbers accessing basic sanitation** | | | **Additional numbers accessing safe sanitation** | | | **Number attending school for an extra year** | **Child deaths averted** | **Maternal deaths averted** |
| --- | --- | --- | --- | --- | --- | --- | --- | --- | --- | --- | --- | --- | --- | --- | --- | --- |
|  |  | All | U5 | Women | All | U5 | Women | All | U5 | Women | All | U5 | Women |  |  |  |
| Andorra | 7,625,863.31 | n/a | n/a | n/a | n/a | n/a | n/a | n/a | n/a | n/a | n/a | n/a | n/a | n/a | n/a | n/a |
| Argentina | 2,427,905,237.00 | n/a | n/a | n/a | n/a | n/a | n/a | n/a | n/a | n/a | n/a | n/a | n/a | n/a | n/a | n/a |
| Aruba | 27,675,410.24 | n/a | n/a | n/a | n/a | n/a | n/a | n/a | n/a | n/a | n/a | n/a | n/a | n/a | n/a | n/a |
| Australia | 3,746,954,009.00 | 0 | 0 | 0 | n/a | n/a | n/a | 0 | 0 | 0 | 766754 | 50521 | 187075 | 3236882 | 161 | 0 |
| Austria | 880,462,981.30 | 0 | 0 | 0 | 0 | 0 | 0 | 0 | 0 | 0 | 1841 | 86 | 437 | 4532 | 0 | 0 |
| Barbados | 120,782,664.10 | n/a | n/a | n/a | n/a | n/a | n/a | n/a | n/a | n/a | n/a | n/a | n/a | n/a | n/a | n/a |
| Belgium | 3,410,099,823.00 | 0 | 0 | 0 | 0 | 0 | 0 | 0 | 0 | 0 | 22907 | 1330 | 5215 | 23461 | 1 | 0 |
| Bermuda | 8,228,747.31 | n/a | n/a | n/a | n/a | n/a | n/a | n/a | n/a | n/a | n/a | n/a | n/a | n/a | n/a | n/a |
| British Virgin Islands | 981,259.07 | n/a | n/a | n/a | n/a | n/a | n/a | n/a | n/a | n/a | n/a | n/a | n/a | n/a | n/a | n/a |
| Brunei | 77,692,529.35 | 0 | 0 | 0 | n/a | n/a | n/a | n/a | n/a | n/a | n/a | n/a | n/a | 247508 | 118 | 0 |
| Canada | 5,129,292,243.00 | 0 | 0 | 0 | 0 | 0 | 0 | 0 | 0 | 0 | 21870 | 1195 | 5258 | 32082 | 9 | 0 |
| Cayman Islands | 151,598.22 | n/a | n/a | n/a | n/a | n/a | n/a | n/a | n/a | n/a | n/a | n/a | n/a | n/a | n/a | n/a |
| Chile | 516,525,621.90 | 12 | 1 | 3 | 159 | 11 | 42 | 213 | 15 | 56 | 15548 | 1094 | 4085 | 7299 | 516 | 1 |
| Croatia | 27,657,867.99 | n/a | n/a | n/a | n/a | n/a | n/a | n/a | n/a | n/a | n/a | n/a | n/a | n/a | n/a | n/a |
| Curaçao | 340,604,430.70 | n/a | n/a | n/a | n/a | n/a | n/a | n/a | n/a | n/a | n/a | n/a | n/a | n/a | n/a | n/a |
| Cyprus | 946,129,851.00 | 0 | 0 | 0 | 0 | 0 | 0 | 0 | 0 | 0 | 12073 | 682 | 3178 | 7926 | 22 | 0 |
| Czechia | 416,179,118.40 | 0 | 0 | 0 | 0 | 0 | 0 | 0 | 0 | 0 | 2848 | 148 | 663 | 3557 | 28 | 0 |
| Denmark | 1,613,722,962.00 | 0 | 0 | 0 | 0 | 0 | 0 | 0 | 0 | 0 | 3885 | 213 | 870 | 7460 | 0 | 0 |
| Estonia | 59,057,975.18 | 0 | 0 | 0 | 0 | 0 | 0 | 0 | 0 | 0 | 946 | 54 | 221 | 544 | 8 | 0 |
| Faroe Islands | 930,989.54 | n/a | n/a | n/a | n/a | n/a | n/a | n/a | n/a | n/a | n/a | n/a | n/a | n/a | n/a | n/a |
| Finland | 813,659,934.40 | 0 | 0 | 0 | 0 | 0 | 0 | 0 | 0 | 0 | 2316 | 128 | 498 | 4077 | 0 | 0 |
| France | 18,174,508,737.00 | 0 | 0 | 0 | 0 | 0 | 0 | 0 | 0 | 0 | 106151 | 6549 | 23658 | 138122 | 7 | 0 |
| French Polynesia | 7,701,005.73 | n/a | n/a | n/a | n/a | n/a | n/a | n/a | n/a | n/a | n/a | n/a | n/a | n/a | n/a | n/a |
| Germany | 31,473,530,131.00 | 0 | 0 | 0 | 0 | 0 | 0 | 0 | 0 | 0 | 135646 | 5744 | 30103 | 150689 | 26 | 0 |
| Gibraltar | 218,752,274.40 | n/a | n/a | n/a | n/a | n/a | n/a | n/a | n/a | n/a | n/a | n/a | n/a | n/a | n/a | n/a |
| Greece | 1,197,341,180.00 | 0 | 0 | 0 | 0 | 0 | 0 | 0 | 0 | 0 | 15980 | 736 | 3607 | 9246 | 11 | 0 |
| Greenland | 1,558,348.01 | n/a | n/a | n/a | n/a | n/a | n/a | n/a | n/a | n/a | n/a | n/a | n/a | n/a | n/a | n/a |
| Hong Kong | 1,449,693,011.00 | 0 | 0 | 0 | 0 | 0 | 0 | 0 | 0 | 0 | 12954 | 441 | 3806 | n/a | n/a | n/a |
| Hungary | 371,718,586.30 | 0 | 0 | 0 | 4 | 0 | 1 | 0 | 0 | 0 | 7869 | 369 | 1858 | 3056 | 46 | 0 |
| Iceland | 48,115,895.28 | 0 | 0 | 0 | 0 | 0 | 0 | 0 | 0 | 0 | 456 | 32 | 109 | 426 | 0 | 0 |
| Ireland | 12,610,075,769.00 | 0 | 0 | 0 | 0 | 0 | 0 | 0 | 0 | 0 | 37117 | 2823 | 9353 | 119558 | 27 | 0 |
| Isle of Man | 233,521,547.80 | n/a | n/a | n/a | n/a | n/a | n/a | n/a | n/a | n/a | n/a | n/a | n/a | n/a | n/a | n/a |
| Israel | 2,064,764,674.00 | n/a | n/a | n/a | n/a | n/a | n/a | n/a | n/a | n/a | n/a | n/a | n/a | n/a | n/a | n/a |
| Italy | 11,123,883,588.00 | 0 | 0 | 0 | 0 | 0 | 0 | n/a | n/a | n/a | n/a | n/a | n/a | 71083 | 7 | 0 |
| Japan | 8,794,704,255.00 | 0 | 0 | 0 | 0 | 0 | 0 | 0 | 0 | 0 | 86628 | 3632 | 17836 | n/a | 59 | 0 |
| Kuwait | 26,525,541.22 | 0 | 0 | 0 | 0 | 0 | 0 | 0 | 0 | 0 | 0 | 0 | 0 | n/a | 0 | 0 |
| Latvia | 57,648,839.88 | 0 | 0 | 0 | 27 | 1 | 7 | 0 | 0 | 0 | 1104 | 57 | 258 | 628 | 17 | 0 |
| Liechtenstein | 53,718,894.03 | n/a | n/a | n/a | n/a | n/a | n/a | n/a | n/a | n/a | n/a | n/a | n/a | n/a | n/a | n/a |
| Lithuania | 90,365,441.13 | 0 | 0 | 0 | 64 | 3 | 16 | 0 | 0 | 0 | 1789 | 89 | 428 | 902 | 28 | 0 |
| Luxembourg | 9,817,480,683.00 | 0 | 0 | 0 | 0 | 0 | 0 | 0 | 0 | 0 | 2675 | 150 | 665 | 41994 | 0 | 0 |
| Macao | 369,735,469.90 | n/a | n/a | n/a | n/a | n/a | n/a | n/a | n/a | n/a | n/a | n/a | n/a | n/a | n/a | n/a |
| Malta | 339,291,601.10 | 0 | 0 | 0 | 0 | 0 | 0 | 0 | 0 | 0 | 3268 | 159 | 743 | 2819 | 16 | 0 |
| Monaco | 386,200.57 | n/a | n/a | n/a | n/a | n/a | n/a | n/a | n/a | n/a | n/a | n/a | n/a | n/a | n/a | n/a |
| Netherlands | 9,273,078,126.00 | 0 | 0 | 0 | 0 | 0 | 0 | 0 | 0 | 0 | 30746 | 1670 | 6972 | 53488 | 5 | 0 |
| New Caledonia | 9,305,628.32 | n/a | n/a | n/a | n/a | n/a | n/a | n/a | n/a | n/a | n/a | n/a | n/a | n/a | n/a | n/a |
| New Zealand | 357,596,900.50 | 0 | 0 | 0 | 0 | 0 | 0 | 0 | 0 | 0 | 2933 | 204 | 713 | 2756 | 6 | 0 |
| Norway | 2,258,743,415.00 | 0 | 0 | 0 | 0 | 0 | 0 | 0 | 0 | 0 | 2153 | 132 | 499 | 10209 | 0 | 0 |
| Oman | 87,111,914.60 | n/a | n/a | n/a | n/a | n/a | n/a | n/a | n/a | n/a | n/a | n/a | n/a | n/a | n/a | n/a |
| Palau | 455.81 | n/a | n/a | n/a | n/a | n/a | n/a | n/a | n/a | n/a | n/a | n/a | n/a | n/a | n/a | n/a |
| Panama | 598,442,087.80 | 3954 | 396 | 1031 | n/a | n/a | n/a | 8101 | 813 | 2114 | n/a | n/a | n/a | 19064 | 1657 | 20 |
| Poland | 2,038,580,211.00 | n/a | n/a | n/a | n/a | n/a | n/a | 17 | 1 | 4 | 63747 | 3230 | 15493 | 20832 | 526 | 0 |
| Portugal | 930,156,451.20 | 0 | 0 | 0 | 0 | 0 | 0 | 0 | 0 | 0 | 13689 | 612 | 3205 | 5604 | 32 | 0 |
| Puerto Rico | 5,145,847.76 | n/a | n/a | n/a | n/a | n/a | n/a | n/a | n/a | n/a | n/a | n/a | n/a | n/a | n/a | n/a |
| Qatar | 103,950,422.60 | 0 | 0 | 0 | 0 | 0 | 0 | 0 | 0 | 0 | 1609 | 81 | 250 | n/a | 0 | 0 |
| San Marino | 4,618,869.10 | n/a | n/a | n/a | n/a | n/a | n/a | n/a | n/a | n/a | n/a | n/a | n/a | n/a | n/a | n/a |
| Saudi Arabia | 2,053,148,878.00 | 0 | 0 | 0 | n/a | n/a | n/a | 0 | 0 | 0 | 60764 | 6157 | 15260 | 40905 | 866 | 5 |
| Seychelles | 149,045,799.50 | n/a | n/a | n/a | n/a | n/a | n/a | n/a | n/a | n/a | n/a | n/a | n/a | n/a | n/a | n/a |
| Singapore | 4,267,824,675.00 | 0 | 0 | 0 | 0 | 0 | 0 | 0 | 0 | 0 | 0 | 0 | 0 | 0 | 895 | 0 |
| Sint Maarten | 4,340,379.59 | n/a | n/a | n/a | n/a | n/a | n/a | n/a | n/a | n/a | n/a | n/a | n/a | n/a | n/a | n/a |
| Slovakia | 372,542,613.80 | 0 | 0 | 0 | 439 | 23 | 111 | 0 | 0 | 0 | 9056 | 471 | 2285 | 3841 | 59 | 0 |
| Slovenia | 190,033,643.90 | 0 | 0 | 0 | 0 | 0 | 0 | 0 | 0 | 0 | 2896 | 148 | 651 | 1425 | 6 | 0 |
| South Korea | 3,514,549,555.00 | n/a | n/a | n/a | n/a | n/a | n/a | n/a | n/a | n/a | n/a | n/a | n/a | n/a | n/a | n/a |
| Spain | 3,914,372,674.00 | 0 | 0 | 0 | 0 | 0 | 0 | 0 | 0 | 0 | 44246 | 2264 | 10676 | 27389 | 43 | 0 |
| Sweden | 2,394,354,159.00 | 0 | 0 | 0 | 0 | 0 | 0 | 0 | 0 | 0 | 6612 | 393 | 1484 | 13807 | 0 | 0 |
| Switzerland | 4,983,650,605.00 | 0 | 0 | 0 | 0 | 0 | 0 | 0 | 0 | 0 | 18537 | 927 | 4454 | 26391 | 1 | 0 |
| Trinidad and Tobago | 233,295,922.60 | 4 | 0 | 1 | n/a | n/a | n/a | 41 | 3 | 10 | n/a | n/a | n/a | n/a | 113 | 0 |
| Turks and Caicos Islands | 13,730,480.18 | n/a | n/a | n/a | n/a | n/a | n/a | n/a | n/a | n/a | n/a | n/a | n/a | n/a | n/a | n/a |
| United Arab Emirates | 929,436,954.90 | 0 | 0 | 0 | n/a | n/a | n/a | 0 | 0 | 0 | 25125 | 1359 | 4722 | 9169 | 101 | 0 |
| United Kingdom | 34,879,939,927.00 | 0 | 0 | 0 | 0 | 0 | 0 | 0 | 0 | 0 | 143211 | 8804 | 33426 | 279646 | 146 | 0 |
| United States | 78,892,848,107.00 | n/a | n/a | n/a | n/a | n/a | n/a | 0 | 0 | 0 | 311256 | 20132 | 72578 | 653184 | 361 | 0 |
| Uruguay | 125,592,509.00 | 7 | 1 | 2 | n/a | n/a | n/a | 9099 | 650 | 2211 | n/a | n/a | n/a | 2424 | 137 | 0 |
| **Total** | | **3977** | **398** | **1037** | **693** | **38** | **177** | **17471** | **1482** | **4395** | **1999205** | **122816** | **472592** | **5283985** | **6061** | **26** |
