# Supplementary material for: Tax abuse—The potential for the Sustainable Development Goals
Source: PLOS Glob Public Health. 2022 Feb 22;2(2):e0000119. doi: 10.1371/journal.pgph.0000119 (PMC10021515; doi:10.1371/journal.pgph.0000119)
Supplement: Attachment — Submitted filename: rebuttal .docx [file pgph.0000119.s013.docx]

PGPH-D-21-00278

Tax abuse - the potential for the Sustainable Development Goals

PLOS Global Public Health

Dear Dr Asante,

Thank you for allowing us the opportunity to submit a revised draft of our manuscript titled “Tax abuse - the potential for the Sustainable Development Goals” to *PLOS Global Health*. We appreciate the time and effort that you and the reviewers have dedicated to providing their valuable feedback and insightful comments. We have been able to incorporate changes to reflect most of the suggestions provided by the reviewers. We have highlighted the changes within the manuscript through the track changes in the submitted document and have listed the changes made below for reference.

**Comments made by the editor**

***1. Authors should provide further details on how tax abuse was quantified, as one of the reviewers pointed out; what they have provided is too sketchy for readers to appreciate the approach/ methodology used.***

We agree with this comment and thank the reviewer for highlighting this. Accordingly, we have included a detailed description of the methodology, including references, used by the Tax Justice Network economists to quantify tax evasion and tax avoidance to achieve a cumulative figure for tax abuse in section 2.1.1 and 2.1.2, lines 157-197.

Tax avoidance

*“Most corporations report the sum of the profits made and costs incurred in different countries as an aggregate of their global operations because they are not required to publicly report this information at the country level. This lack of transparency affords the opportunity to move profits out of the countries with a higher tax rate (often where the actual business is done) and into countries with low tax rates, thus avoiding paying tax. While many members of the Organisation for Economic Co-operation and Development (OECD) require multinational corporations with revenues over 750 million Euros to report the profit they make and the tax they pay in each country, this is done privately. However, in 2020, the OECD began to publish their members’ data. The report included information on the country in which the profit was made, the amount of tax paid, the number of tangible assets and employees, and summarised information such as the number of corporations reporting.(28) There are, however, limitations including lack of information on the ownership structure of corporations or details on intra-company transactions.(29)*

*Analysis of this OECD data was carried out by economists in the Tax Justice Network to estimate corporate tax avoidance for 215 countries using methodologies previously reported.(30–32) Tax avoidance was assessed using the country-by-country reporting datasets to calculate the mismatch between expected and reported profits, with the former assessed using figures on labour and sales. Profit misalignments was calculated as the difference between expected and reported to provide estimates on the levels of tax avoidance within each country with data. (33)”*

Tax evasion

*“When wealthy business owners deposit their revenues in tax havens, profits are often simultaneously deposited within these banks, leading to an artificial decrease in tax payments and a loss of tax in the individuals’ home country. These types of tax abuses are much more complex to quantify due to a lack of data, as these types of tax abuses are illegal. The Tax Justice Network Tax applied an approach based on methodologies by Henry (34), Zucman (35), and Alstadsaeter, Johannesen, and Zucman to estimate the amount of deposits which are used to evade tax.(36) First, abnormal deposits were identified by pinpointing jurisdictions which had an abnormally high foreign deposit rate in relation to the size of the country’s economy. Inward bank deposits are highly correlated with GDP (when countries with high levels of bank secrecy are excluded) which allows abnormal deposits to be identified and traced to their origin countries. Abnormal deposits combined with existing estimates of total global offshore wealth from Alstadsaeter, Johannesen, and Zucman (36) were used to derive the values of offshore wealth from each country of origin. Using the effective tax rate in each country, these figures were used to calculate the losses in income tax revenue for each country.(33)”*

***2. Should explore alternative ways of presenting the results; too many tables are used.***

We agree that the amount of information presented in the tables may be overwhelming and have therefore removed Tables 1, 2, and 3.

Table 1 had presented the data availability for each variable. We have removed this as readers can see which countries have been included in the appendix.

Tables 2 and 3 had presented the results by country. We have moved them to the appendix rather than remove them entirely as we feel these will be of interest to the readers, especially for those living in those countries. We are happy to remove them from the appendix if space is a concern and signpost the reader to the results on our website. In addition we have included a country case study on Mozambique as an example in the results in section 3, in lines 243-253.

***3. Data sources should be indicated under the tables.***

We have inserted the data sources under each of the retained tables in the manuscript.

***4. What sensitivity analysis was conducted to check the robustness of the results?***

This is a great point, thank you. We have included a detailed breakdown of the peer-reviewed econometric modelling of the GRADE tool in section 2.2 (see lines 198-220).

To answer the question about checking the robustness of the results we have added the following and inserted a reference which includes a link to the actual and modelled estimates which can be found in Mendeley data (see Section 2.2, lines 218-221).

*“A comparison of the actual and fitted results used by the GRADE modelling confirms how close the modelled estimates are to the actual coverage of the SDG variables, which confirms the robustness of the model and the precision of the results presented here.”*

***5. The terms 'tax abuse' and 'tax loss' should be clarified - tax abuse may be a deliberate act to avoid/evade tax which is illegal, tax loss may result from legitimate tax concessions granted to companies by governments.***

We have introduced these terms in Section 1.4, lines 133-140 and defined them:

*“Sometimes multinational corporations use artificial tax-avoiding arrangements to move profits from a high to a low tax jurisdiction to minimise the tax due. This is usually described as tax avoidance and is legal.(26) On the other hand, tax evasion, which may be carried out by both private individuals and multinational corporations, is illegal. However, relative to tax avoidance, tax evasion is much smaller especially in low and lower-middle-income countries, where tax avoidance comes to 5.5.% of tax revenue and tax evasion comes to 0.3% of tax revenue (see box 2). However, as both negatively impact government revenues, public services, and the SDGs, which are fundamental economic and social human rights, (27) we consider these under the umbrella term of 'tax abuses', and hereafter refer to both under this term..”*

This reviewer raises the issue of legitimate tax concessions or tax incentives, which are waivers granted by governments, but waivers or incentives are not tax abuses. We have clarified this with the following line in Section 1.3, lines 121-122:

*“As a result, governments compete to attract foreign investment and offer tax incentives, which are not considered tax abuses but do reduce tax revenue.”*

***6. Clarify whether this study is focused on multinational corporations only (one reviewer raised this issue). Note that local companies also evade tax.***

Thank you. The answer is yes because the data on tax avoidance analysed by the Tax Justice Network comes from the OECD database on country by country reports which come from multinational corporations. See Section 2.1.1, lines 162-168.

*“While many members of the Organisation for Economic Co-operation and Development (OECD) require multinational corporations with revenues over 750 million Euros to report the profit they make and the tax they pay in each country, this is done privately. However, in 2020, the OECD began to publish their members’ data. The report included information on the country in which the profit was made, the amount of tax paid, the number of tangible assets and employees, and summarised information such as the number of corporations reporting.(28).”*

And lines 169-170:

*“Economists in the Tax Justice Network analysed this OECD data to estimate corporate tax avoidance for 215 countries using methodologies previously reported.(30–32)”.*

The data on tax evasion on the other hand identifies abnormally high foreign deposits relative to the size of country’s economy which are then traced to the origin country. Tax evasion may by carried out by both private individuals as well as multinational corporations but relative to tax avoidance are much smaller especially in low income countries. For example in the case of lower- income countries tax avoidance comes to 5.5.% of tax revenue whereas tax evasion comes to 0.3% of tax revenue.

We have further justified this decision in our literature review in section 1.3, lines 113-118:

*“Experts believe that increasing revenue from corporate income tax is the most feasible short to medium term solution to financing public services and corporate tax contributes much more (about 13%) in low-income countries compared to high income countries (about 7%).(24) Businesses are already operating within the boundaries of low- and lower-middle-income countries, meaning that a change in tax policy could provide a short-term process to broaden the tax base.(19) Therefore, our analysis focuses on how the revenue generated from multinational corporations can drive progress towards the SDGs.”*

We referred to this decision in our limitations in section 4.2, lines 300-303:

*“Additionally, the figures from the Tax Justice Network focus on multinational corporations, meaning that tax losses by smaller businesses are not regarded. However, we justify this as a unified taxation system for multinational businesses could have a significant and immediate impact on revenue in lower income countries (17).”*

***7. It would have been helpful to provide some information on corporate tax rates and tax incentives/concessions in LMICs as part of the background material.***

We have inserted section 1.3 on Taxation in low- and lower-middle income countries. We have included the contribution of corporate income tax and include this line which highlights the importance of corporate taxes to low-income countries in lines 113-115:

*“Experts believe that increasing their revenue from corporate income tax is the most feasible short to medium term solution to financing public services and corporate tax contributes much more (about 13%) in low-income countries compared to high-income countries (about 7%)* (8)*.*

We have also clarified that tax waivers are not tax abuses but rather a result of a decision of a government togrant special incentives to attract foreign direct investment. Given this comment, we do include a brief discussion on tax incentives and their impact on lower income countries in Section 1.3:

*“The purposes of taxes include revenue generation to pay for public services, the redistribution of wealth, and deter unhealthy behaviours, such as taxes on tobacco. (21) Taxes are a significant form of revenue in low- and lower-middle-income countries, constituting 70% of total revenue. (22) However, government revenue in these countries is very low. They face enormous challenges broadening their tax base, such as increasing income tax or enlarging the formal sector. (23)*

*Experts believe that increasing revenue from corporate income tax is the most feasible short-term solution to financing public services. Corporate tax contributes much more (about 13%) to low-income countries' tax revenue than high-income countries (about 7%). (24) Multinational corporations are already operating within the boundaries of low- and lower-middle-income countries, and changes to global tax policy could provide a quick way to increase tax revenues. (19) Therefore, our analysis focuses on the potential for additional revenue from multinational corporations to drive progress towards the SDGs.*

*Ideally, governments design their tax regimes and expenditure to benefit the public welfare. However, they must also shape policies to attract investment as the private sector is the leading provider of employment and capital. As a result, governments compete to attract foreign investment and offer tax incentives, which are not considered tax abuses but reduce tax revenue. Whilst tax incentives have become commonplace for these reasons, there is a debate if they benefit low-income countries. The revenue leaks from incentives and other tax exemptions offered to multinational corporations significantly impact governments' ability to provide essential services and, thus, fulfil human rights obligations. Additionally, governments often compensate for the revenue shortfall resulting from exemptions by raising taxes that impact ordinary citizens, for example, value-added taxes on goods or services. (20) While tax incentives have been empirically linked to corruption and may negatively impact economic activity, (21) governments still offer them out of fear that higher tax rates will deter investors. (19) This competition has been commonly dubbed 'the race to the bottom’ and poses a significant threat to sustainable economic development and the progress towards the SDGs. “*

The term tax abuses does not include tax incentives or waivers which are often granted by governments to multinational companies with the aim of attacting foreign direct investments.

**Comments by reviewer 2**

***1. While the study does well to discuss the pathways in the background, there was little effort to discuss the context of the study. It will be good to have some background statistics to show the extent of the problem, at least across the income levels. For instance, what is the current state of sanitation and water access in the regions? What about tax revenues and evasion? This will help place the study into some better context.***

We have inserted a discussion of the current progress towards the SDGs in section 1.1, lines 42-76:

*“Health is determined by multiple factors provided both within and outside health facilities, such as water, sanitation, and education. Half of the reduction in mortality between 1990-2015 resulted from increased coverage of these health determinants. (1,2) Because of the impact of these determinants of health on survival, achieving higher coverage of water, sanitation, education, and healthcare belong to the Sustainable Development Goals, a set of goals agreed by the global community through the United Nations to drive international development and increase global equality.(3) Safe water, safe sanitation, and education are Sustainable Development Goals (SDGs) 4 and 6. The SDG 4 and 6 targets are to ensure that all children complete free primary and secondary education and all have access to safe water and safe sanitation by 2030. Accessing these determinants of health would, in turn, reduce child and maternal mortality rates and contribute to progress towards SDGs 3 and 5 (see box 1 and figure 1).(4) SDG 3 aims to reduce the under-five mortality (U5M) to less than 25 per 1000 live births and the maternal mortality ratio (MMR) to less than 70 per 100,000 live births in all countries by 2030. SDG 5 aims to -increase gender equality, including universal access to reproductive healthcare and sexual education, which are significant determinants in the reduction of maternal mortality. Hereafter we refer to these health determinants by the relevant SDG, remembering that achieving these SDGs are a matter of economic and social human rights,(5) and currently a substantial number of countries are off track to achieving these goals.*

*There has been significant progress towards SDG 6, with two billion individuals gaining access to safe drinking water and 2.4 billion to safe sanitation between 2000 and 2020. However, many still lack access to water and sanitation. In 2020, two billion people lacked access to safe water and 771 million to basic water, half of the latter being in Sub-Saharan Africa. 3.6 billion individuals lacked access to safe sanitation, of which 1.7 billion lacked basic sanitation and 494 million practiced open defecation. 9 out of 10 of those practicing open defecation live in Eastern and South Eastern Asia and Sub-Saharan Africa. (6) These levels of inequality are also apparent within progress towards SDG 4 in the increase of access to education. 88% of all children complete secondary education in North America and Europe, compared to only 29% in Sub-Saharan Africa, where only 62% of children complete primary education. (7) Progress in 53 countries is off-track to achieve the desired child survival rates, of which two thirds are in Sub-Saharan Africa. (8) In 2017, 86% of the women dying during childbirth from preventable complications were in Sub-Saharan Africa and Asia. (8) Sub-Saharan Africa has the highest rates of maternal and under-5 mortality rates, with 1 in 37 women dying during childbirth in 2020 (compared to 1 in 7800 in Australia and New Zealand) and 76 out of 1000 children, or 1 in 13 children, dying before the age of five in 2019 (compared to 1 in 264 in Australia and New Zealand). (9) Low and lower-middle income regions, particularly Sub-Saharan Africa, are at risk of not reaching the SDGs by 2030, so we must look towards progressive measures to accelerate progress over the next decade and reduce the inequality gap between nations. Increasing government revenue through raising tax revenue could provide the fundamental change that is necessary to reduce this gap.”*

We have also further elaborated upon tax abuses across the regions in section 1.4 lines 147-148:

*“Most strongly affected, on the other hand, are Africa and Latin America, losing an annual $26 Billion (7% of tax revenue) and $43 Billion (4.2% of tax revenue) to tax abuse respectively. (18)”*

***2. The techniques used in this paper are not common. It is therefore important that emphasis is placed on providing sufficient detail to the reader. At the moment this is not the case. The approach to estimate the impact of tax abuse is very nuanced. There should be a systematic discussion on the procedure and approach to arrive at each of the estimates explained in the results.***

We have included more detailed descriptions both of the methodologies used by the Tax Justice Network to estimate tax abuses (see Section 2.1 and comment 1 by editor) and the GRADE modelling, which was used to estimate the impact of an increase in government revenue on the variables used within this study (see Section 2.2).

*“To estimate the impact of an increase in government revenue equivalent to the tax abuse in each country on progress towards SDGs 3, 4 , 5 and 6 (increasing survival, access to basic drinking water/sanitation/education, and increasing gender equality), we employed economic modelling from the Government Revenue and Development Estimations project (the GRADE).(12) The GRADE uses government revenue data from the UNU Wider database (36) and data on the SDGs (basic/safe drinking water, basic/safe sanitation, school years and under-5/maternal survival rates) from the World Development Indicators (37). and several decades of data from 217 countries to model the impact of government revenue on progress towards the SDGs and includes governance indicators from the World Governance Indicators, including political stability and absence of violence, government effectiveness, regulatory quality, the rule of law, and control of corruption (38). (see Appendix for details on variables). The estimates are realistic because it is assumed that governments spend any additional income in the same way they have been in recent years rather than being reallocated to a specific sector (39). A comparison of the actual and fitted results confirms how close the modelled estimates are to the actual coverage of the SDG variables, which confirms the robustness of the model and the precision of the results presented here (40).”*

***3. In line with the previous comment, it will be also be helpful to have a clear definition of the variables.***

See previous comment (specifically refer to lines 205-206). Please also see the Appendix for a detailed breakdown of the variables used in the GRADE.

***4. Form the explanation provided on tax abuse, it appears the paper only captures tax avoidance and not tax evasion. If this is the cases, kindly clarify. Another concern with the measure of tax abuse is that it appears to only focus on multinationals. This is not quite clear in the paper. If this is the case, it will be good to limit the extent to which results are generalized. It will also be good to acknowledge the limitation.***

Our paper uses the term tax abuses includes both tax evasion and tax avoidance. Please refer to Sections 1.4, 2.1.1 and 2.1.2 for changes made (see comments above, e.g. comment 5 by editor). Also see our Textbox 2, which elucidates this further.

We have added an elaboration that this study only focuses on multinationals in section 2.1. We have justified this decision in our literature review in section 1.3 and referred to this decision in our limitatinos in section 4.2.

***5. Again, in the estimation of the impact, it is not clear if the tax loss provides for each of the facilities jointly or separately. For instance, in a single country, will that tax loss provide for water, sanitation and schooling jointly? This is not clear. Would be good to discuss these in detail in the methodology.***

Thank you for your comments. The methodology does not take the costs for the provision of individual SDG targets and equate these to the additional revenue. Rather, the GRADE methodology uses data over many decades and models how a change in government revenue has impacted the provision of water/sanitation/education and thus each of the SDG targets jointly. Thus the model allows a prediction of how an increase in revenue would impact the SDG targets. The model incorporates six dimensions of governance and makes no assumptions about how the additional revenue is spent but rather predicts the imapct based on what has happened in the past. We have elaborated this further in Section 2.2.

***6. Also, are the deaths averted as a result of the water and sanitation provided?***

Yes, we elaborated this more clearly in section 1.1 (lines 49-50) and referred to Figure 1, which visually demonstrates the relationship between government revenue, the provision of water/sanitation/education, and mortality rates.

*“Accessing these determinants of health would, in turn, reduce child and maternal mortality rates and contribute to progress towards SDGs 3 and 5 (see box 1 and figure 1).(4)”*

Thus the models allows the prediction of how an increase in revenue would impact the SDG targets including safe water and sanitaiotn,education and survival, so it is reasonable to assume that the deaths averted are associated with the increased access to the critical determinants of health.

We also highlighted this again in Section 3, lines 245-250:

*“We estimate that if there were an increase in government revenue equivalent to the tax abuse over a ten year period, on average, 572,388 people (of which 99,140 are children and 134,577 are women) would have access to basic drinking water, on average 724,449 people (of which 124,914 are children and 170,450 are women) would have access to basic sanitation, and 63,222 children would attend school for an additional year. These increases would influence the survival rates (see figure 1), and 22,879 under-5 and 3,671 maternal deaths would be averted with this increase in revenue.”*

***7. In computing the figures for access to basic facilities, did you use cost of each of the facilities within countries? How exactly were these numbers arrived at?***

Please see answer to comment 5 above.

The methodology does not does not take the costs for the provision of individual SDG targets and equate these to the additional revenue. Rather, the GRADE methodology uses data over many decades and models how a change in government revenue has impacted the provision of water/sanitation/education and thus each of the SDG targets jointly. Thus the models allows the prediction of how an increase in revenue would impact the SDG targets. The model incorporates six dimensions of governance and makes no assumptions about how the additional revenue is spent but rather predicts the imapct based on what has happened in the past. We have elaborated this further in Section 2.2.

***8. The presentation of the tables can be improved. In the current form, there is so much information in each table. Also, there are too many tables presented without texts to explain each table.***

We agree that the amount of information presented in the tables may be overwhelming and have therefore removed Tables 1, 2, and 3. To replace Table 1, which presented data availability for each variable, we have inserted a sentence at the end of the first paragraph in Section 3 to summarise availability of data. To replace Tables 2 and 3, which included country-by-country breakdowns of the results and data used, we have included an example of how we conducted our analysis in section 3. We have also included the tables in the appendix and referenced the working paper on the GRADE website, which includes these tables.

**Comments by reviewer 1**

**1*. I suggest that the authors add more information in the Discussion and conclusion.***

We have expanded our conclusion to include limitations, implications, and recommendations, see Section 5.

*“Tax abuse contributes to millions not accessing their rights and hinders progress towards the SDGs. The limitations of this study include not having data for abuses by smaller businesses, longer time periods, or for indirect losses. These limitations indicate that the figures presented within this paper are likely underestimated. Most global tax abuse can be attributed to four enabling countries: the United Kingdom, the Netherlands, Switzerland, and Luxembourg. The four countries enabling more than half of global tax abuses, alongside other key economic players, should review their tax policies to ensure they are meeting their human rights obligations and are not negatively impacting government revenue and the progress towards the SDGs. To reduce tax abuse and increase survival rates, governments should publicly publish the disaggregated, company-level data to assist the assessment of the changes needed to the global tax system. Curtailing tax abuse would contribute substantially to progress towards the SDGs and result in many accessing their fundamental economic and social rights*.”

We have also extended our discussion to include corruption and why we focused on multinational corporations, see Section 4.2, lines 300-303:

*“Additionally, the figures from the Tax Justice Network focus on multinational corporations, meaning that tax losses by smaller businesses are not regarded. However, we justify this as a unified taxation system for multinational businesses could have a significant and immediate impact on revenue in lower income countries (20).”*

Also in Section 4.1, lines 282-290:

*“Corruption is often cited as a cause of lost revenue. A small number of developed countries, such as the UK, contribute to a large proportion of this increase. However, the GRADE takes a country’s history of corruption and associated revenue leaks into account when modelling the impact of revenue on progress towards the SDGs. As an increase in government revenue has been shown to be associated with an improvement in the quality of governance over time, it is vital to ensure that preventable leaks caused by developed countries such as tax abuses are curbed, particularly as a small number of developed countries, such as the UK, contribute to a large proportion of tax abuses. Therefore, focusing on increasing revenue through curtailing leaks to tax abuse will allow countries to develop effective governance and democratic governments that can ensure human rights are met and the SDG targets can be met by 2030..”*

**2. *A papragraph or two about the challenges of corruption in achieving SDGs.***

Thank you for your suggestion. We have also included a paragraph about corruption in the discussion (see previous answer) and have referenced this in Section 1.2 of the manuscript. The GRADE modelling includes the six dimensions of governance, one of which is Control of Corruption, therefore if a country is very corrupt an increase in revenue will have less impact and the model reflects this. If, of course, further measures were taken to reduce corruption, this would amplify the impact of an increase in government revenue and further progress towards the SDGs. Additionally, Baskaran and Bigsten empirically show than an increase in fiscal capacity can actually reduce corruption and we refer to this in section 1.2, lines 98-102:

“*Corruption is often highlighted as a significant source of revenue leakage within low and lower-middle-income countries. However, a study of 31 African countries showed that a 1% increase in tax to GDP ratio reduces corruption by 0·08 points (measured on a scale of 0-6).(18) To summarise, an increase in government revenue accelerates progress towards the SDGs and may also strengthen governance, which would further amplify this effect.”*

**3. *The determinants of health need to be clearly spelled out***

We have added further elaboration of the determinants of health and how they relate to the SDGs. We have referred to these by their respective SDG from this point onward , see Section 1.1. lines 42-56:

*“Health is determined by multiple factors provided both within and outside health facilities, such as water, sanitation, and education. Half of the reduction in mortality between 1990-2015 resulted from increased coverage of these health determinants. (1,2) Because of the impact of these determinants of health on survival, achieving higher coverage of water, sanitation, education, and healthcare belong to the Sustainable Development Goals, a set of goals agreed by the global community through the United Nations to drive international development and increase global equality.(3) Safe water, safe sanitation, and education are Sustainable Development Goals (SDGs) 4 and 6. The SDG 4 and 6 targets are to ensure that all children complete free primary and secondary education and all have access to safe water and safe sanitation by 2030. Accessing these determinants of health would, in turn, reduce child and maternal mortality rates and contribute to progress towards SDGs 3 and 5 (see box 1 and figure 1).(4) SDG 3 aims to reduce the under-five mortality (U5M) to less than 25 per 1000 live births and the maternal mortality ratio (MMR) to less than 70 per 100,000 live births in all countries by 2030. SDG 5 aims to increase gender equality, including universal access to reproductive healthcare and sexual education, which are significant determinants in the reduction of maternal mortality. Hereafter we refer to these health determinants by the relevant SDG, remembering that achieving these SDGs are a matter of economic and social human rights,(5) and currently a substantial number of countries are off track to achieving these goals.”*

**4. *The method of identification of sources of data and how exclusion criteria. I would suggest a method commonly used in published/secondary data***

We have added a detailed explanation of why we used the State of Tax Justice Report in Section 2.13. The State of Tax Justice Report is the first of its kind and uses figures provided by the OECD. We have also provided the following to justify our choice in Section 2.1.3, Lines 191-197:

*“We conducted secondary data analysis on this report as firstly, it is the first of its kind to provide a country-by-country breakdown of total tax abuse, and secondly, based on the sources and methodologies described above, we found that data was collected in an appropriate manner to assure its validity and reliability. Additionally, reliability of secondary data can be verified if similar methodologies produce similar results (34). The $427 billion figure annual global tax abuse is in tandem with figures provided by others ($500-600 billion) (27–29), but slightly lower, indicating that these figures are conservative.”*

**5. (in reference to conclusion) *This needs more information. Make clear what the conclusion is, and what the recommendations and limitations***

See comment 1.

We have expanded our conclusion to include limitations, implications, and recommendations. We have also extended our discussion to include corruption and why we focused on multinational corporations.

**6. *Talk about corruption in low income countries with respect to tax***

See reviewer 2, comment 2 above.

With respect to the association between corruption and tax, Zelekha and Sharabi have empirically demonstrated that countries with high levels of tax incentives also have high levels of corruption. (10) We have included this in our literature review in Section 1.3.

**7*. Include how these tax revenue allocations will be undertaken to ensure value for money is achieved.***

Please see answer to reviewer 2, comment 5 above.

The GRADE methodology uses data over many decades and models how a change in government revenue has impacted the provision of water/sanitation/education and thus each of the SDG targets jointly. Thus the models allows the prediction of how an increase in revenue would impact the SDG targets imapct based on what has happened in the past. The model makes no assumptions about how the additional revenue is allocted as this would be unrealistic and each country makes their allocation decisions based on their own priorities. We have elaborated this further in Section 2.2.

**8. *Issues of equity in the allocations are not explicitly addressed in the model.***

Please see answer to reviewer 2, comment 5 above.

The GRADE methodology makes no assumptions about how the additional revenue is allocated as this would be unrealistic and each country makes their allocation decisions based on their own priorities and political economy.

We have also made the following changes as we had mistakenly deflated the total tax abuse values from 2020 to 2010. However, after discussing with the Tax Justice Network, whilst the figures were marked as 2020 figures, the values for tax avoidance were derived from 2017 and the figures on tax evasion were from 2016. We have deflated the values individually and accordingly reran these through the GRADE and updated the tables. This is elaborated in Section 2.3, lines 223-225:

*“Figures on tax avoidance were used from the OECD dataset in 2016 were used for all countries apart from the US, where a more complete data set from 2017 was used. Figures on tax evasion are from 2018. We converted tax abuse estimates from the year of their origin to 2010 figures as the GRADE uses constant 2010 USD.”*

Due to these changes, we have removed the column on total tax abuse in 2020 USD from the data tables, as we would otherwise have to insert additional columns. Given the already large amount of data, we felt that this information would be superfluous. We justify this as the data on total tax abuse (evasion and avoidance) from their year of origin are readily available from the State of Tax Justice report. We only present our deflated figures within the manuscript.

We also made the following changes:

- Removed the term “human rights” from the title of Section 1.1, as this does not accurately reflect the contents of the section
- Restructured section 1.4 “Tax abuse and government revenue” due to inserting section 1.3 on “Taxation in low- and lower-middle-income countries”. Section 1.4 also now more accurately frames international tax abuses as presented within our study and sets tax abuses in LICs and LMICs within this context (see lines 145-148).
- Inserted Section 4.1 “Research in context” to summarise our findings and set them more generally within the wider context.

We believe these additional changes contribute towards making our paper a higher quality output and support readers in understanding our research.

We look forward to hearing from you in due time regarding our submission and to respond to any further questions and comments you may have.

Sincerely,

Marisol Lopez
